# National Trauma Registries in LMICs: Long-Overdue Priority: Comment on "Neurotrauma Surveillance in National Registries of Low- and Middle-Income Countries: A Scoping Review and Comparative Analysis of Data Dictionaries"

**DOI:** 10.34172/ijhpm.2023.7504

**Published:** 2023-04-29

**Authors:** Zerubabbel K. Asfaw

**Affiliations:** Department of Neurosurgery, Icahn School of Medicine, New York City, NY, USA

**Keywords:** Neurotrauma, Injury Data Set, Global Neurosurgery, Trauma Registry, Low- and Middle-Income Countries

## Abstract

The burden of trauma-related mortality is inversely related to income on an individual and national scale. Barthélemy et al highlight the significant variation of neurotrauma data included in national injury registries of low- and middle-income countries (LMICs) when compared to the World Health Organization (WHO) minimal dataset for injury (MDI). Moreover, the authors emphasize that the non-existence and underutilization of nationally standardized trauma registries hinder the data-driven identification of factors contributing to neurotrauma and subsequent attempts to improve neurotrauma care. Establishing a nationally standardized trauma registry should be prioritized by all stakeholders involved in curbing trauma-related mortality and building research capacity in LMICs. In this commentary, previous successful efforts to establish and maintain robust registries in LMICs through local and international partnerships are highlighted. The lessons and challenges chronicled in establishing such registries can inform future efforts to implement a nationally standardized trauma registry.

## The Global Disparity in Trauma Burden

 Injuries cause 8% of global mortality — 4.4 million deaths — and 10% of all years lived with disability. The top 3 causes of death for individuals aged 5-29 are all injury-related, namely road-traffic accidents (RTAs), homicides, and suicides.^1^ Nearly 90% of injury-related deaths occur in low- and middle-income countries (LMICs), which continue to suffer significant health and economic consequences from such deaths than mortality due to HIV/AIDS, malaria, and tuberculosis combined.^2^ The death rate due to RTA in LMICs is 27.5 per 100 000 relative to 8.3 per 100 000 in high-income countries (HICs).^3^ Previous reports indicate that RTA is expected to become the third leading cause of global mortality ahead of ischemic heart disease by 2030.^4^ In light of such dire projections, the establishment of national trauma registries remains imperative for determining factors that contribute to neurotrauma and subsequent attempts to improve neurotrauma care and outcomes in LMICs. This commentary will highlight three international collaborations that can inform future initiatives that seek to establish national trauma registries.

## The Current State of Neurotrauma Surveillance in LMICs

 Brain injury is a leading cause of injury-related deaths, with RTA accounting for 50% of all traumatic brain injuries.^5^ LMICs struggle significantly to curb mortality due to critical health resource scarcity and low neurosurgical workforce density. Previous studies indicate that the median number of neurosurgeons per 100 000 is 0.03 for LMICs and 0.97 for HICs, with some LMICs lacking a neurosurgical training program.^6^ In some LMICs with a neurosurgery workforce, most neurosurgeons reside in urban centers that are not easily accessible to the country’s rural population.^7^ Although regional collaborations have boosted neurosurgical workforce density, the nascent research capacity in most LMICs has limited the characterization of the actual neurotrauma burden in these countries.

 LMICs have to maximize the potential benefit that can be obtained from each critical healthcare resource. Data derived from nationally standardized trauma registries can be utilized to determine which sectors in the continuum of neurotrauma care would significantly improve patient outcomes with adequate resource investment. Barthélemy et al reviewed the literature on national trauma registries and reported that nine countries had a publicly-available database.^5^ There was a significant variation among the reviewed national trauma databases in terms of staff responsible for data collection, the inclusion of data points pertinent to neurotrauma, and tools for data collection and storage. A non-randomized convenience sampling of contacts and websites of ministries of health yielded national registries of a few more countries. Among the 16 registries examined by Barthélemy et al, all contained at least 6 of the 8 recommended neurotrauma data points from the World Health Organization (WHO) minimal dataset for injury (MDI). On a global scale, only 29 of the 115 countries reporting health statistics to WHO had a comprehensive national trauma registry.^5,8^ The authors emphasized that the non-existence or underutilization of nationally standardized trauma registries significantly impeded research capacity-building efforts and yielded an immense neurotrauma data disparity across nations with different income levels.^5^ As an illustration, Barthélemy et al mentioned that the WHO global burden of disease study of 2016 indicated that certain LMICs in sub-Saharan Africa had lower rates of neurotrauma than countries in North America and Europe with more healthcare resources, including well-established health data collection systems.^5,9^

## Establishing National Trauma Registries for Robust Neurotrauma Surveillance

 The global neurotrauma data disparity has multifactorial underpinnings, which include low neurotrauma workforce density and distribution, lack of significant investment in infrastructural and skilled personnel requirements for national registry development and maintenance, inadequate healthcare policy geared towards setting up health data collection systems, and the immense clinical volume handled by the few practicing neurosurgeons and other specialized clinical workforces in LMICs.^5^ While addressing all of the above factors requires a long-term multipronged approach, Barthélemy et al suggested utilizing the WHO international registry for trauma and emergency — a free web-based platform that incorporates validated MDI to collect patient-level clinical data — on a national scale is an ideal first step. Equally important, the authors emphasized the need to standardize trauma registry data collection not only among centers in a given country but also across different nations.^5^ Current regional organizations, such as the College of Surgeons of East Central and Southern Africa, that focus on developing surgical capacity in LMICs through a standardized training program may facilitate the standardization of trauma registries among member countries. Implementing a standardized trauma registry on a global scale can catalyze international research collaborations and codification of health policies based on more conclusive data. Barthélemy et al noted that some countries or centers might lack an electronic medical record system. As a result, a nationally standardized trauma registry should allow for a multimodal data collection approach while maintaining a high standard for the inclusion of the greatest amount of patient data as well as data quality and validation. The WHO Trauma System Maturity Index and the Evaluation Framework for Injury Surveillance Systems were cited by the authors as tools that have been employed for assessing data quality and validation in LMIC trauma systems and registries.^5^

 Odukoya et al reported the feasibility and challenges of setting up an HIV-associated cancer project registry in Nigeria using the Research Electronic Data Capture (REDCap) system.^13^ The authors noted that the bidirectional partnership between institutions in Nigeria and USA yielded a successful installation and configuration of a REDCap database that caters to the needs of the Nigerian institution. The choice of REDCap over other database systems was due to the system’s availability free of charge to all non-profit organizations who can sign an end-user agreement with Vanderbilt University. Moreover, the ubiquitous use of REDCap — 4705 institutions in 139 countries — also makes it ideal for transferring data to collaborating institutions and finding troubleshooting tips for problems encountered in various settings.^13^ Odukoya et al noted that a considerable hurdle was a lack of trained and experienced personnel. Therefore, the Nigerian staff were effectively trained in managing a REDCap database through virtual workshops, the annual REDCap conference, and interactions with fellow REDCap users via the global REDCap consortium. Due to limited internet connectivity, the authors prepared a pdf version of data entry forms that were later uploaded in batches to the web format of the database by personnel trained in data entry. Redundancies were built into the data entry, assessment, and validation workflow to ensure data quality. To mitigate the lack of continuous electrical power that interrupts data entry and quality check tasks, equipment that provides an uninterruptible power source for 5 hours was connected to the main server. Regarding data collection from sites located in remote locations, the authors emphasized that a virtual private network solved any issues pertinent to connectivity and access to the REDCap server. While Odukoya et al acknowledged the challenges involved in building research capacity in the absence of an infrastructure optimized for data collection, they presented a compelling case for the feasibility of setting up context-specific registries in LMICs using existing resources and replicable methodologies.^13^

 In a similar study, Choi et al demonstrated that healthcare institutions in LMICs can establish and maintain a registry that provides valuable data for modifying practice paradigms.^14^ The authors set up a database based on the Vermont-Oxford Network to collect data points pertinent to neonatal care. The main challenges in maintaining the database were data collection and quality assessment due to the lack of dedicated personnel. As a result, data entry was jeopardized when clinicians and other stakeholders involved in data collection were under significant clinical duty burden or no longer participated in the initiative. However, Choi et al noted that, despite such challenges, the registry led to quality improvement initiatives and clinical outcomes research.^14^ Similar to Odukoya et al, the authors acknowledged that establishing a REDCap-based data collection system and training key stakeholders in data entry would have addressed the notable challenges encountered in the endeavor.^13,14^ The neonatal database was also reproduced in a context-specific and upgraded format in other LMICs, such as Kenya and Ethiopia. Although the database established in Rwanda was limited to a single institution, it illustrates that such registries are feasible and replicable.^14^

 In another collaboration between institutions in Nigeria and the United States of America, Aliyu et al outlined the necessary components of building infrastructure capacity for research administration and management in LMICs.^15^ The authors pointed out the need for bilateral administrative engagement across institutions as well as community engagement in research conducted by LMIC institutions. Moreover, the report highlighted that building research administration and management capacity must be coupled with adequate research ethics training. The authors emphasized that research derived from registries in LMICs can be aligned with local values and cultural perspectives by establishing a community advisory board. The salient tenets of establishing and maintaining a high-quality registry in LMICs, as outlined by Aliyu et al, can serve as a template for other regional and global efforts to initiate a sustainable growth of research capacity in LMICs.^15^

 National trauma registries are necessary for uncovering the actual neurotrauma burden in LMICs.^2,5^ Even though establishing such registries remains challenging in LMICs, the paucity of resources makes a nationally standardized registry much more imperative for the optimal allocation of healthcare resources.^14^ Neurosurgeons and other stakeholders engaged in global neurosurgery should incorporate building national trauma registries as a principal component of capacity-building endeavors. The lessons and challenges reported in developing databases in LMICs can inform future efforts to implement a nationally standardized trauma registry in LMICs.

## Ethical issues

 Not applicable.

## Competing interests

 Author declares that he has no competing interests.
